# Emergence of mature cortical activity in wakefulness and sleep in healthy preterm and full-term infants

**DOI:** 10.1093/sleep/zsy096

**Published:** 2018-05-14

**Authors:** Kimberley Whitehead, Maria Pureza Laudiano-Dray, Judith Meek, Lorenzo Fabrizi

**Affiliations:** 1 Department of Neuroscience, Physiology and Pharmacology, University College London, London, United Kingdom; 2 Elizabeth Garrett Anderson Obstetric Wing, University College London Hospitals, London, United Kingdom

**Keywords:** quiet sleep, active sleep, neonatal, REM sleep, non-REM sleep, θ, δ, β, visual, postnatal age

## Abstract

**Study Objectives:**

Cortical activity patterns develop rapidly over the equivalent of the last trimester of gestation, in parallel with the establishment of sleep architecture. However, the emergence of mature cortical activity in wakefulness compared with sleep states in healthy preterm infants is poorly understood.

**Methods:**

To investigate whether the cortical activity has a different developmental profile in each sleep–wake state, we recorded 11-channels electroencephalography (EEG), electrooculography (EOG), and respiratory movement for 1 hr from 115 infants 34 to 43 weeks–corrected age, with 0.5–17 days of postnatal age. We characterized the trajectory of δ, θ, and α-β oscillations in wakefulness, rapid eye movement (REM) sleep, and non-REM sleep by calculating the power spectrum of the EEG, averaged across artifact-free epochs.

**Results:**

δ-Oscillations in wakefulness and REM sleep decrease with corrected age, particularly in the temporal region, but not in non-REM sleep. θ-Oscillations increase with corrected age in sleep, especially non-REM sleep, but not in wakefulness. On the other hand, α-β oscillations decrease predominantly with postnatal age, independently of sleep–wake state, particularly in the occipital region.

**Conclusions:**

The developmental trajectory of δ and θ rhythms is state-dependent and results in changed cortical activity patterns between states with corrected age, which suggests that these frequency bands may have particular functional roles in each state. Interestingly, postnatal age is associated with a decrease in α-β oscillations overlying primary visual cortex in every sleep–wake state, suggesting that postnatal experience (including the first visual input through open eyes during periods of wakefulness) is associated with resting-state visual cortical activity changes.

**Statement of Significance**

This study is the first to separately define the developmental trajectory of neural oscillations during wakefulness and sleep, using a cohort of 115 healthy infants. Our large sample allows us to compare the development of brain rhythms in wakefulness—which is scarce and therefore difficult to record in the newborn period—to rapid eye movement (REM) and non-REM sleep. We show that changes in the δ and θ band are state-dependent. Meanwhile, the decrease in α-β rhythms overlying visual cortex with postnatal age is independent of state. Our results help us to build a model of the interaction between intrinsic brain maturation and environmental factors across the sleep–wake cycle. Future work could use learning paradigms to further investigate the way that cortical oscillations may be modulated by experience.

## Introduction

Cortical activity patterns develop rapidly over the equivalent of the last trimester of gestation [1, 2]. Neural oscillations can be used as an index of cortical network maturation as they reflect the assembly of mature functional networks and predict structural brain growth [3, 4]. They thereby offer a clinically valuable window onto brain development [2]. There are 3 frequency bands which have been consistently used as markers of neonatal cortical maturation: δ, α-β, and θ oscillations. δ-Oscillations are customarily considered as an index of immature brain activity [5–7]. α-β rhythms are also associated with immature cortical activity patterns [2, 8, 9]. On the other hand, θ oscillations are a marker of mature brain activity [5, 8, 10–12].

Periods of wakefulness, rapid eye movement (REM) sleep, and non-REM sleep are first associated with characteristic neural activity patterns from 31 to 34 weeks–corrected age (CA) as sleep–wake architecture emerges [13, 14], although wakefulness is still extremely scarce, occupying as little as 2 per cent of preterm life [15]. In preterm infants, non-REM sleep is always associated with an alternating electroencephalography (EEG) pattern (tracé alternant), but from 37 weeks–CA, an additional slow-wave EEG pattern begins to emerge [16, 17]. Finally, by 40 weeks–CA, cyclical periods of wakefulness are well-organized around feeds on demand and electrographic correlates of sleep–wake state are clearly defined [1, 18].

Although the emergence of organized periods of wakefulness is a landmark developmental milestone, it is unknown whether the emergence of mature cortical rhythms in the neonatal period differs in wakefulness compared with sleep states, because wakefulness has been little studied [12, 19–22]. In adults, wakefulness and sleep states are each associated with different cortical patterns [23–26]. For example, wakefulness is characterized by α activity which is thought to be associated with cognitive capacity [27], whereas non-REM sleep is associated with spindle activity which plays a role in memory consolidation [28]. These segregated cortical functions are likely to play a complementary role, for example information experienced during wakefulness is efficiently consolidated as memory when the brain is “off-line,” i.e. sleeping [29].

Little is also known about the maturation of cortical activity in healthy preterm infants at low risk of adverse neurodevelopment, because in previous studies the effect of postnatal age (PNA) was often associated with, and therefore confounded by, very preterm birth and long-term intensive care admission [30]. Therefore, within a well-defined normative neonatal cohort, it will be easier to distinguish the separate effects of (1) CA and (2) PNA.

To investigate the normative emergence of mature oscillatory activity in wakefulness and sleep states across the neonatal period, and which factors influence this, we recorded resting EEG in a cross-sectional cohort of 115 healthy infants aged 34 to 43 weeks–CA with a PNA between 0.5 and 17 days and characterized the effect of CA and PNA on δ, θ, and α-β oscillations for each state separately: wakefulness, REM sleep, and non-REM sleep.

## Methods

### Participants

One hundred fifteen infants were recruited from the postnatal ward and special care ward at the Elizabeth Garrett Anderson wing of University College London Hospitals between July 2015 and July 2016 (Table 1) for research EEG examination. No infants required EEG for clinical purposes. No neonates were acutely unwell, receiving neuroactive medication (including caffeine), or respiratory support at the time of study. All neonates were neurologically normal both at the time of study and at the date of discharge, and were considered at low risk of adverse neurodevelopment, based on the review of medical notes and the discharge summary. Cranial ultrasound scans were reported as normal when participants were referred for one (*n* = 40). All EEGs were assessed as normal for CA by a clinical scientist (K.W.) according to standard criteria, and presence of appropriate sleep architecture including REM-onset sleep, and transition of the slow wave to tracé alternant EEG pattern during non-REM sleep [31–33].

**Table 1.** Demographics

*n* = 115 infants	
Median (range) corrected age (weeks + days)*	38 + 6 (34 + 0 – 43 + 1) Preterm CA: *n* = 38; Term CA: *n* = 77
Median (range) postnatal age (days)	3 (0.5–17)
Sex	47.8% female
Birth weight (grams) (range)	2880 (1550–4320)
Multiple births	14.8%
Ward location	73.9% postnatal; 26.1% special care^†^

*CA is defined as gestational age at birth plus postnatal age. For example, an infant born at 35 weeks + 2 days, who is 3 days old, is corrected age 35 weeks + 5 days. Term is defined as ≥37 weeks CA [33].

^†^Infants on the postnatal ward are cared for by their parents; infants on the special care ward require low-intensity nursing care.

## Table 1: Demographics

Ethical approval was obtained from the NHS Research Ethics Committee, and informed written parental consent was obtained prior to each study. Separate written parental consent was obtained to publish the photographs. The study conformed to the standards set by the Declaration of Helsinki guidelines.

### Recording setup

Resting EEG was recorded for approximately 1 hr, in line with recommended best practice [34], between 09:00 and 17:30 hr. Recording electrodes (disposable Ag/AgCl cup electrodes) were placed overlying frontal (F4, F3), central (C4, Cz, C3), mid-temporal (T8, T7), posterior-temporal (P8, P7), and occipital cortex (O2, O1), positioned according to the modified international 10/20 electrode placement system. On 6/115 occasions, a reduced number of electrodes (up to two less) were used because the infant became slightly unsettled. The reference electrode was placed at Fz and the ground electrode was placed at FC6/5. Target impedance was <10 kΩ [1]. A single lead I ECG was recorded from both shoulders. The respiratory movement was monitored with an abdominal transducer and electrooculography (EOG) was recorded using electrodes positioned laterally to the eyes. We have previously shown that this polygraph setup does not cause stress to infants, in a comparable cohort [35]. EEG was recorded with a direct current (DC)-coupled amplifier from DC-800Hz using the Neuroscan (Scan 4.3) SynAmps2 EEG/EP recording system. Signals were digitized with a sampling rate of 2 kHz and a resolution of 24 bit.

### EEG preprocessing

Data analysis was carried out using EEGLAB v.13 (Swartz Center for Computational Neuroscience), custom-written Matlab code, and IBM SPSS version 22. Mains interference was removed with a 50 Hz notch filter (4th order Butterworth filter) and, for each epoch, a baseline correction was used to remove DC offset. Recordings from electrodes which had poor contact with the scalp were rejected. Missing and discarded recordings were then estimated with spherical interpolation as implemented in EEGLAB. Recordings were reviewed and periods classified into wakefulness, REM sleep, and non-REM sleep (subclassified as tracé alternant or slow-wave EEG pattern) [31, 32] (Supplementary Table S1 for a summary of criteria used and Supplementary Figure S1 for illustrative examples). Sections of indeterminate sleep were discarded from further analysis. As not all infants cycled through all the sleep–wake states during the recording period, wakefulness, REM sleep, non-REM sleep: tracé alternant pattern, and non-REM sleep: slow wave pattern were obtained in 38/115, 108/115, 79/115, and 37/115 test occasions, respectively. We assessed whether the incidence of sleep–wake states captured varied according to the CA or PNA of the infants with a binary logistic regression using the Enter Method. In line with previous reports that tracé alternant is gradually replaced by the slow-wave pattern, the likelihood of the slow-wave pattern of non-REM sleep being captured doubled with every week of CA, whereas the likelihood of the tracé alternant pattern of non-REM sleep being captured slightly diminished (*p* ≤ .005; Exp(B) slow-wave = 2.06, Exp(B) tracé alternant = .78) [36, 37]. On the other hand, the likelihood of wakefulness being captured slightly increased with PNA (*p* = .014; Exp(B) = 1.26). We analyzed the tracé alternant pattern of non-REM sleep, alongside REM sleep and wakefulness, because it was present in more infants in line with other studies [5], and the developmental trajectory of the two EEG patterns of non-REM sleep was comparable (Supplementary Results).

### EEG analysis

Twelve second artifact-free epochs, during which the infants were not undergoing any stimulation, were extracted from each of the sleep–wake state sections. This is an appropriate epoch length because it contains over two cycles of our lowest frequency of interest (0.2 Hz) [38]. The median number (interquartile range) of epochs included for each infant was as follows: wakefulness = 7 (2–14), REM sleep = 17 (7–32), and non-REM sleep = 16 (9–33). The power spectrum (µV^2^) was calculated for each channel and epoch, using a Hanning window to reduce spectral leakage. This was then averaged across all of the epochs within a sleep–wake state per infant, leading to a single power spectrum per sleep–wake state per participant.

The power in slow δ (0.2–2 Hz), θ (4–6Hz), and α-β (8–20 Hz) frequency bands was then extracted for the channels overlying midline central, right and left frontal, central, mid-temporal, posterior-temporal, and occipital cortex (Supplementary Figure S2) (right and left channels for lateral sites averaged, after checking that there was no statistically significant difference in the power between hemispheres). We evaluated the influence of CA and PNA on the power of each frequency band with multivariable linear regression modeling using the Stepwise Method. Our model used the power of each frequency band, for every region in every state, as the dependent variable with two possible explanatory variables: CA and PNA. We report β weights (standardized regression coefficients) for each model so that, in the case of a model in which the power of a frequency band is related to both CA and PNA, the two independent variables can be directly compared to determine the predominant factor (β weights indicate by how many standard deviation units the dependent variable will change for one standard deviation change in the independent variable).

Throughout data are plotted for the posterior-temporal and occipital regions because our key findings relate to these areas, whereas the results for all regions are reported in the text. When both PNA and CA were associated with the power of a frequency band, we provide a visual representation of the interaction between these influences by plotting the power of the frequency band against CA for lower PNA (0.5–2 days; median: 2 days) and higher PNA (3–17 days; median: 5 days) subgroups.

We next investigated whether there was a redistribution of the frequency content between sleep–wake states with CA. We assessed this by analyzing power in each frequency band at every region and testing for an interaction between sleep–wake state and CA group (preterm vs. term) using a two-way analysis of variance (ANOVA). We confirmed our findings by performing the same tests in a subgroup of 21 infants (preterm *n* = 9; term *n* = 12) who had cycled through all three sleep–wake states during the recording, using a repeated measures ANOVA so that states could be compared within-participant. The Huynh–Feldt correction method was used if the assumption of sphericity was not met by the data.

To control for the risk of a type I error due to multiple comparisons, statistical significance threshold was set to 0.01 for all tests.

## Results

### Results: δ power decreased with CA in wakefulness and REM sleep

**Figure 1.**
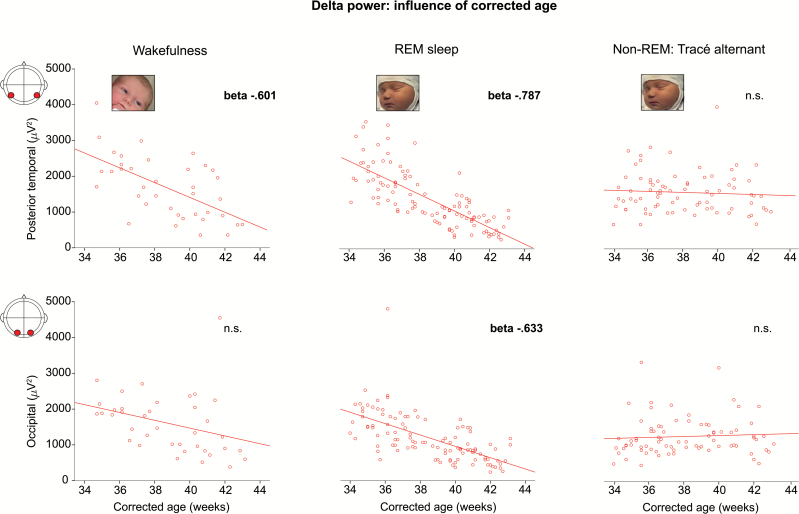
δ-Power decreases with increasing corrected age in wakefulness and REM sleep, but not in non-REM sleep. Scatter plot of δ power over the posterior-temporal and occipital regions against corrected age for each participant, and line of best fit. β-Values for the linear regression are reported only when significant (*p* < .01).

δ-Power decreased with CA in wakefulness and REM sleep, but not in non-REM sleep (Figure 1). In wakefulness, this decrease was specific to the temporal region (posterior-temporal: *R*^2^ = .361, β = −.601; mid-temporal: *R*^2^ = .216, β = −.465; model fits *p* ≤ .003). In REM sleep, this decrease was again more pronounced over the posterior-temporal area, but also widespread (posterior-temporal: *R*^2^ = .610, β = −.787; all other areas except frontal and midline central: *R*^2^ = .174–.401, β = −.418 to −.633; model fits *p* < .001).

The developmental profile of δ power was largely independent of PNA, which only contributed slightly to the decrease in δ power over the posterior-temporal area in REM sleep (introduction of PNA significantly improves the *R*^2^ of CA-only model [*p* = .007] although PNA β was just −.171, two-factor model fit *p* <.001, and no collinearity between CA and PNA: *r* = −.139; *p* = .075).

In non-REM sleep, there was a slight increase in δ power with CA, specific to the midline central region (*R*^2^ = .084, β = .290, model fit *p* = .009).

### Results: θ power increased with CA in sleep

**Figure 2.**
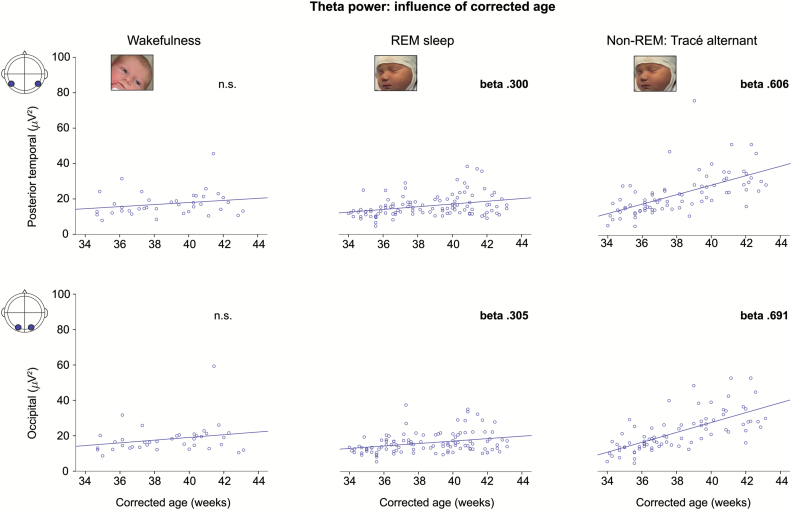
θ-Power increases with corrected age in non-REM and REM sleep, but not in wakefulness. Scatter plot of θ power over the posterior-temporal and occipital regions against corrected age for each participant, and line of best fit. β-Values for the linear regression are reported only when significant (*p* < .01).

θ-Power was associated with an opposite developmental profile compared with δ power, increasing with CA only in sleep and especially non-REM sleep for every region (non-REM sleep: *R*^2^ = .218–.478, β = .467–.691, model fits *p* <.001 for every region; REM sleep: *R*^2^ = .062–.107, β = .221–.312, model fits *p* ≤ .009 for every region except frontal) while unassociated with PNA (Figure 2).

### Results: Occipital α-β power decreased with PNA in every state

**Figure 3.**
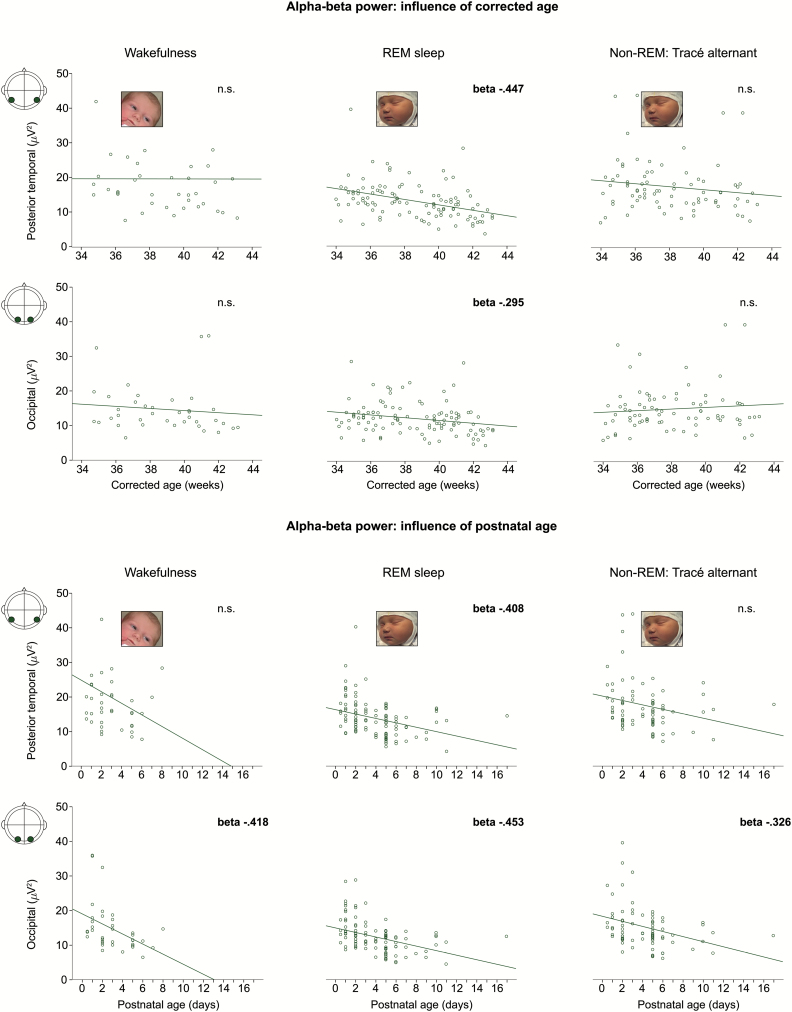
α-β Power decreases with increasing postnatal age in every sleep–wake state, but only with increasing corrected age in REM sleep. Scatter plot of α-β power over the posterior-temporal and occipital regions against corrected age (upper panel) and postnatal age (lower panel) for each participant, and line of best fit. β-Values for the linear regression are reported only when significant (*p* < .01). One outlier dataset not shown in the wakefulness posterior-temporal scatterplots: corrected age = 41 weeks, 103 µV [2].

**Figure 4.**
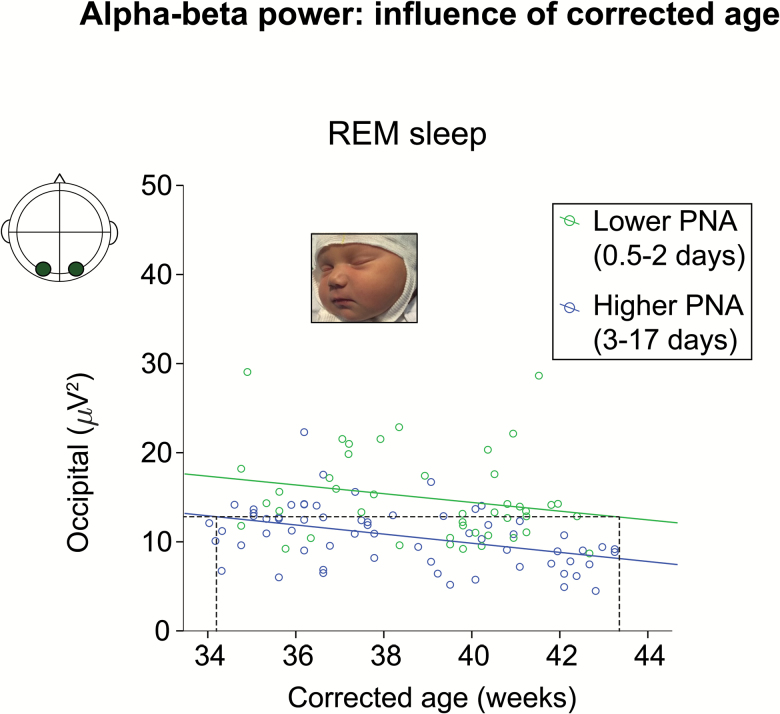
Occipital α-β power in REM sleep decreases with corrected age but infants with a higher PNA reach lower power values at an earlier corrected age. Scatter plot of α-β power over the occipital region against corrected age for participants with lower (green, *n* = 44, median = 2 days) and higher (blue, *n* = 64, median = 5 days) PNA, and lines of best fit for the two groups. The dashed lines represent an example to show that infants of higher PNA had an α-β power value of 13 μV^2^ at 34 weeks corrected age, whereas infants of lower PNA did not reach this value until 43 weeks corrected age.

α-β Power resembled δ power by decreasing with CA in REM sleep, but decreased most steeply with PNA for every region except mid- and posterior-temporal (*R*^2^ with both factors: .162 and .334, PNA β = −.311 to -.453, CA β = −.195 to −.519, model fits *p* < .001) (Figures 3 and 4). Figure 4 illustrates the additive effect of PNA and CA on occipital α-β activity in REM sleep: power decreased with higher CA but lower power values were reached at an earlier CA for infants with a higher PNA.

The decrease in occipital α-β power with PNA was also present in wakefulness and non-REM sleep, the only developmental finding seen in every sleep–wake state (*R*^2^ ≥ .106, PNA β = −.326 to −.453, model fits *p* ≤ .009).

### Results: δ and θ power was redistributed between sleep–wake states with CA

**Figure 5.**
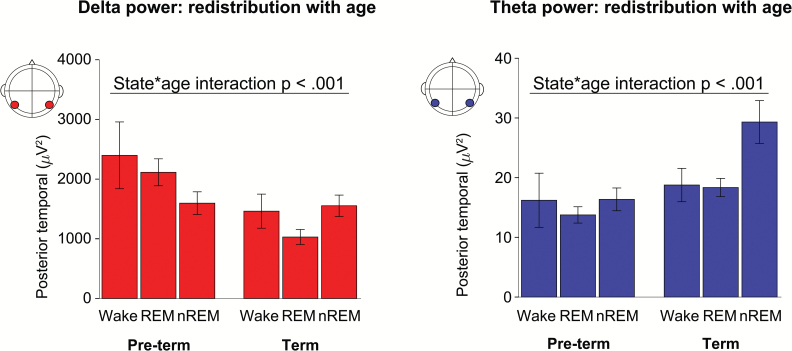
δ- and θ-Power is redistributed between sleep–wake states with corrected age. Mean δ power (left panel) and θ power (right panel) across sleep–wake states in corrected preterm and term infants. Error bars = 95% confidence intervals; Wake = wakefulness; REM = REM sleep; nREM = non-REM sleep.

After showing that there were state-specific changes in δ and θ power with CA, we investigated whether this resulted in a redistribution of power between sleep–wake states with CA (Figure 5). δ and θ Power was redistributed between states from preterm to term age for mid- and posterior-temporal and occipital regions (state*age group interaction, *p* < .001), which was more widespread for θ power (frontal and central regions, *p* < .001) (Figure 5). This redistribution of power with CA could also be appreciated in a subgroup of 21 infants who had cycled through all three states during the recording (i.e. using a within-participant comparison of states) (state*age group interaction mid-temporal region: δ *p* = .006, θ trend *p* = .015). In line with the minimal effect of CA on α-β power, there was no redistribution of power between states with CA in that frequency band. All results are summarized in graphical form in Supplementary Figure S3.

## Discussion

Our results show that the developmental trajectories of δ and θ activity are state-dependent: δ activity declines in wakefulness and REM sleep, and θ activity increases in non-REM sleep. Meanwhile, the decrease in α-β oscillations overlying visual cortex is mostly associated with PNA in every sleep–wake state.

### State-dependent developmental trajectory of δ oscillations

Our data confirm previous findings that δ activity decreases with CA [5–7] and show that this is specific to wakefulness and REM sleep, whereas delta activity in non-REM sleep persists at a similar level, or slightly increases. δ-Activity is proposed to mediate memory formation and synaptic pruning during non-REM sleep [29, 39], offering a possible explanation of why δ rhythms in non-REM sleep are maintained across development: the capacity to form memories is essential throughout life from the newborn period onwards [40].

We demonstrate that this decrease in δ activity is most pronounced in the posterior-temporal region, indicating that this area may be relatively immature and then undergo particularly dramatic preprogrammed maturation towards the equivalent of the last trimester of gestation. Experiments using simultaneous EEG and functional magnetic resonance imaging (fMRI) show that posterior-temporal δ rhythms in preterm infants are associated with insula (i.e. association cortex) activity, and these δ oscillations decrease as the insula enters a phase of accelerated maturation [41]. Therefore, decreasing posterior-temporal δ rhythms could indicate maturation of association cortex towards the end of the equivalent of gestation. This is in line with evidence that sensory networks are relatively mature by late preterm age, whereas association cortices are still developing [42, 43]. Our data suggest that declining δ activity with CA in REM sleep is a normative feature because, in a neonatal cohort at high risk of adverse neurodevelopment, δ activity increased with age in REM sleep [44].

Previous reports have noted that the “delta brush” pattern (δ + over-riding α-β rhythms) declines more prominently in wakefulness and REM sleep than non-REM sleep towards the equivalent of the end of the gestation [2]. Our data suggest that this decrease in δ brushes during wakefulness may be largely under-pinned by declining δ rhythms, as only δ rhythms, but not α-β rhythms, decline with CA in wakefulness. A maturational decrease in δ activity during wakefulness may be related to early exploratory behavior, as animal models have associated δ rhythms during wakefulness with impaired motor functioning [45].

### State-dependent developmental trajectory of θ oscillations

Our data confirm previous studies that θ activity increases with CA [5, 8, 10–12], and show that this is specific to sleep, especially non-REM sleep, and not present for wakefulness. As θ activity facilitates synaptic plasticity, with long-term potentiation organized around the phase of the θ wave [46], its increasing power could reflect expanding sleep-specific brain functions. In adults, θ activity is prominent in REM sleep but extremely scarce in non-REM sleep [24, 47, 48]. This pattern is reversed in our neonatal cohort, emphasizing the need for models which interpret the functional role of neural oscillations within a developmental framework.

Overall, non-REM sleep shows a unique developmental profile compared with the other sleep–wake states with increasing θ activity and maintained δ activity (Figure 5). This age-related divergence of cortical activity patterns between non-REM sleep on the one hand, and wakefulness and REM sleep on the other, could reflect the emergence of specific cortical functioning in non-REM sleep.

### State-independent developmental trajectory of α-β oscillations overlying visual cortex

One of the novel findings of the present study is that PNA, more than CA, is specifically associated with a decrease in α-β oscillations in healthy infants. Supportive that α-β oscillations are sensitive to PNA, a previous study of very preterm infants found that only oscillations in the α-β range were significantly reduced in every sleep–wake state in those infants of greater PNA [49]. Spontaneous electrical activity in the α-β range is considered a marker of cortical immaturity because animal models have shown that it has a permissive, and perhaps instructive, role in neural circuit development, and it is prominent in early human life [2, 8, 9, 50]. Our data suggest that extra-uterine experience (PNA) is particularly associated with the decrease of this immature brain rhythm.

In particular, we show that visual cortex is the only region in which this decrease is present for wakefulness, REM, and non-REM sleep. This regional specificity suggests that postnatal experience may be especially associated with resting-state visual network activity changes, in line with evidence that postnatal experience predicts the development of cortical binocularity and gaze following [51, 52]. One explanation for this is that visual input through open eyes only occurs postnatally such that birth marks a profound expansion in visual experience: the eyes remain shut until birth, whereas, after preterm or full-term birth, 2–4 per cent of neonatal life is spent with eyes open (in wakefulness) [15, 18, 53]. In fact, we show that the incidence of wakefulness increases with PNA. Visual scanning during eyes-open wakefulness can evoke “lambda” waves overlying visual cortex in newborn infants, demonstrating that the extra-uterine eyes-open state influences visual cortical functioning [1, 54]. The association of visual experience with α-β activity would be in line with a mouse model in which α-β oscillations are prominent on the first day after eye opening but poorly defined after this [55].

This study has some limitations. In particular, we did not capture every sleep–wake state in every infant, resulting in uneven sample sizes for each state. This could have reduced our power to detect subtle differences in the developmental trajectories of cortical activity during these states. Although we have a relatively limited range of PNA, it would not have been possible to extend our PNA range without including some infants at high risk of adverse neurodevelopment, as such infants tend to be hospitalized for longer, and this would have undermined our aim to study normative development.

### Summary

In summary, our data from 115 healthy preterm and full-term infants provide a model of the emergence of differentially state-specific neural activity patterns in newborn infants. Crucially, existing models of sleep–wake architecture—which rely on oscillatory markers such as sleep spindles and α rhythms which do not emerge until 2–3 months of age [32, 56]—are not applicable to the newborn brain. By characterizing the development of cortical activity in each sleep–wake state in a large normative neonatal cohort, we offer a window onto the developing brain and the neurobiology which underpins environmental influences [57].

## Supplementary Material

Supplementary material is available at *SLEEP* online.

Supplementary Table S1

Supplementary Figure S1

Supplementary Figure S2

Supplementary Figure S3

Supplementary Material
