# Supplementary material for: Emergence of mature cortical activity in wakefulness and sleep in healthy preterm and full-term infants
Source: Sleep. 2018 May 14;41(8):zsy096. doi: 10.1093/sleep/zsy096 (PMC6093466; doi:10.1093/sleep/zsy096)
Supplement: Supplementary Table S1 [file zsy096_suppl_table_s1.docx]

Table S1: Criteria used to define sleep-wake state (adapted from references ^31,32^ in main text)

| **Vigilance state** | **Respiration** | **Behaviour** | **EEG** |
| --- | --- | --- | --- |
| Wakefulness | Irregular | Continuous or almost continuous wide-open eyes; sustained elevated EMG tone; spontaneous limb and mouth movements | Continuous, low to medium voltage |
| REM sleep | Markedly irregular: differences in rate and depth of ventilation, brief periods of relatively decreased / increased respiratory rate | Eyes closed; rapid eye movements; sucking; facial grimaces; jaw jerks; limb twitches; chin, body and limb tremors; intermittent  stretching | Continuous, mainly low to medium voltage |
| Non-REM sleep: slow- wave pattern | Regular | Eyes closed; scant body movements except for generalized myoclonic “startles” | Nonstop high-voltage |
| Non-REM sleep: tracé alternant pattern |  |  | Alternating: high voltage bursts alternate with lower amplitude periods |
| Indeterminate sleep | Eyes closed, but respiratory, behavioural and EEG features are discordant and do not permit definite assignment to a sleep state | | |
