# Supplementary material for: Emergence of mature cortical activity in wakefulness and sleep in healthy preterm and full-term infants
Source: Sleep. 2018 May 14;41(8):zsy096. doi: 10.1093/sleep/zsy096 (PMC6093466; doi:10.1093/sleep/zsy096)
Supplement: Supplementary Figure S1 [file zsy096_suppl_fig_s1_resubmit.pdf]

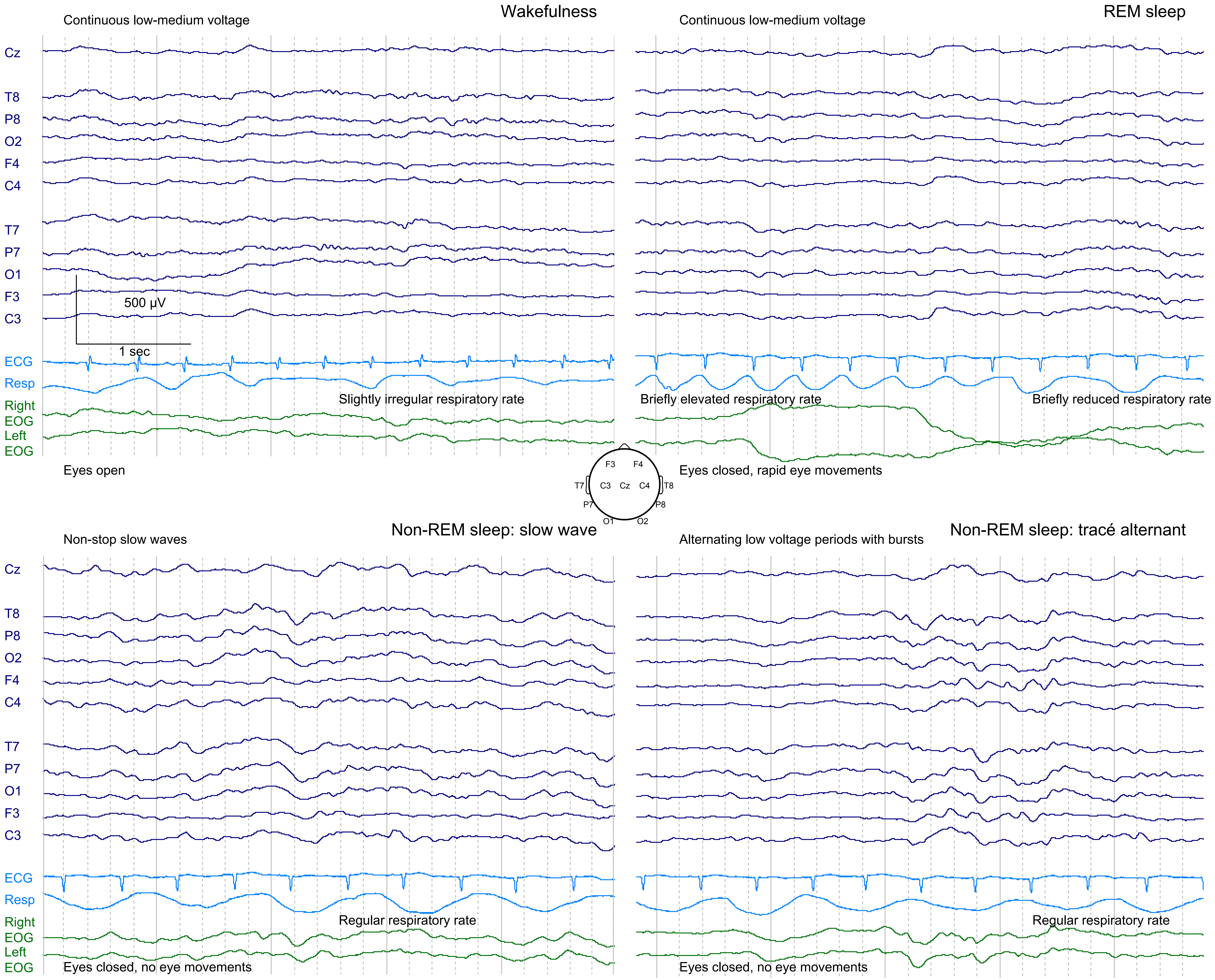

Wakefulness
Continuous low-medium voltage
Cz
T8
P8
O2
F4
C4
T7
P7
O1
F3
C3
500 μV
1 sec
ECG
Resp
Slightly irregular respiratory rate
Right EOG
Left EOG
Eyes open
REM sleep
Continuous low-medium voltage
Briefly elevated respiratory rate
Briefly reduced respiratory rate
Eyes closed, rapid eye movements
F3
F4
T7
C3
Cz
C4
T8
P7
P8
O1
O2
Non-REM sleep: slow wave
Non-stop slow waves
Regular respiratory rate
Eyes closed, no eye movements
Non-REM sleep: tracé alternant
Alternating low voltage periods with bursts
Regular respiratory rate
Eyes closed, no eye movements
