# Supplementary figures and images for: Emergence of mature cortical activity in wakefulness and sleep in healthy preterm and full-term infants
Source: Sleep. 2018 May 14;41(8):zsy096. doi: 10.1093/sleep/zsy096 (PMC6093466; doi:10.1093/sleep/zsy096)

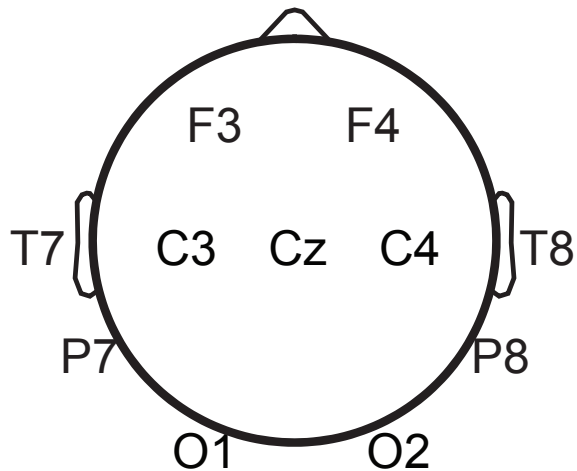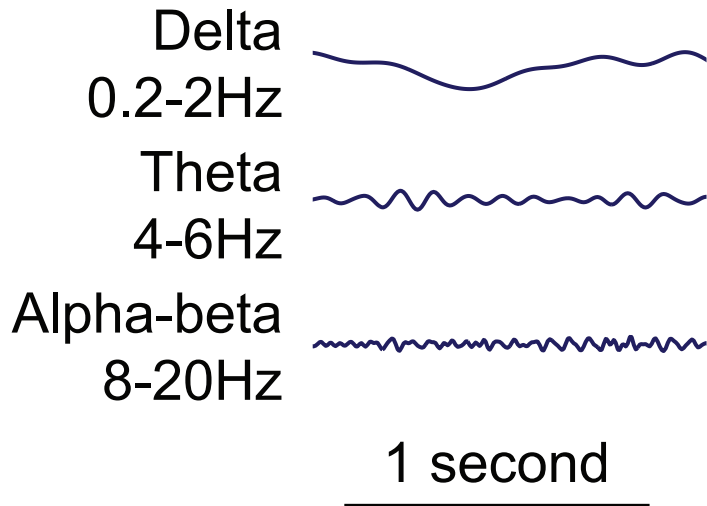

Supplement: Supplementary Figure S2 [file zsy096_suppl_fig_s2_resubmit.pdf]
