# Supplementary material for: Emergence of mature cortical activity in wakefulness and sleep in healthy preterm and full-term infants
Source: Sleep. 2018 May 14;41(8):zsy096. doi: 10.1093/sleep/zsy096 (PMC6093466; doi:10.1093/sleep/zsy096)
Supplement: Supplementary Figure S3 [file zsy096_suppl_fig_s3_resubmit.pdf]

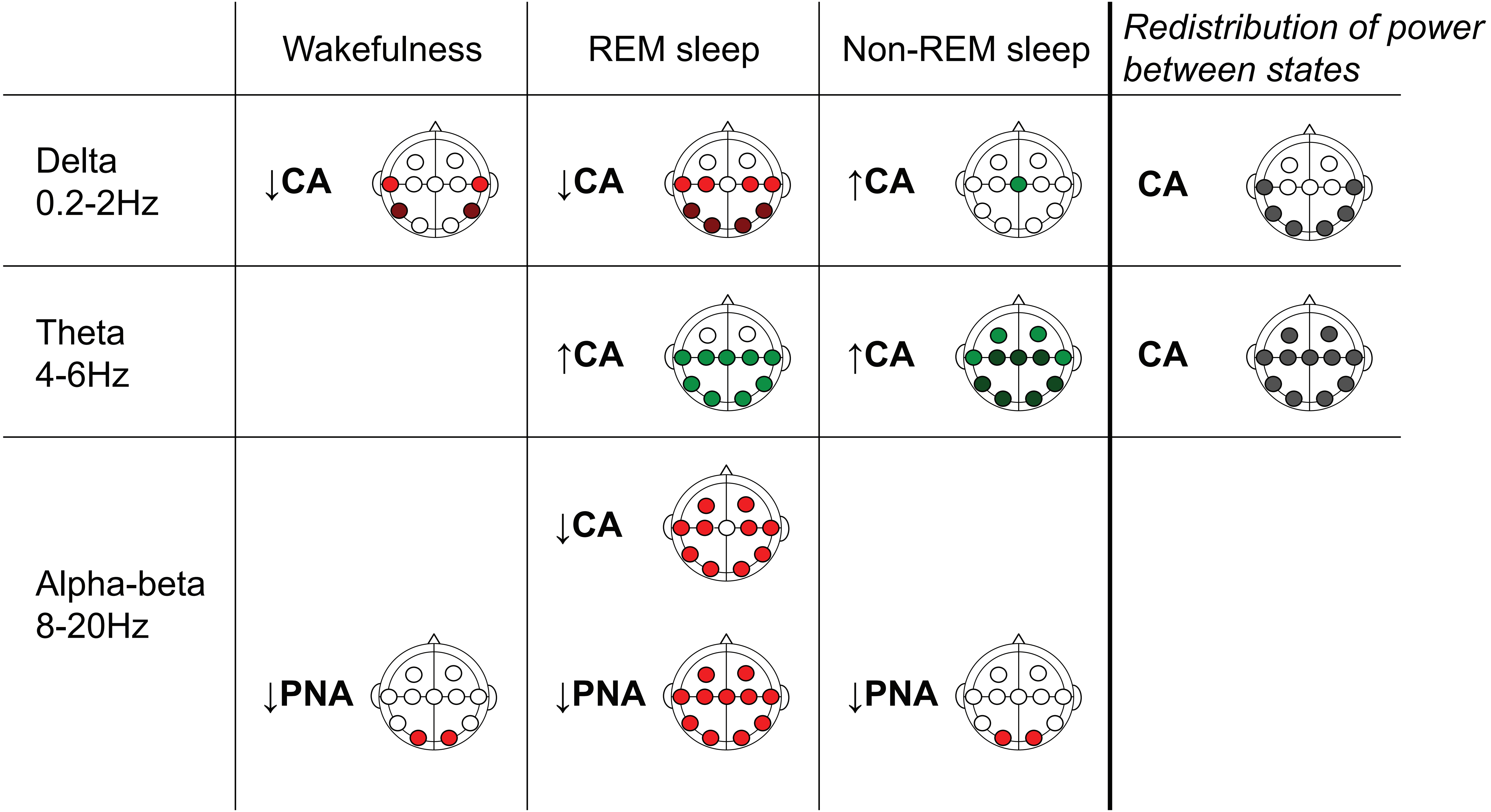
Wakefulness
REM sleep
Non-REM sleep
Redistribution of power between states
Delta
0.2-2Hz
↓CA
↓CA
↑CA
CA
Theta
4-6Hz
↑CA
↑CA
CA
Alpha-beta
8-20Hz
↓CA
↓PNA
↓PNA
↓PNA
