# Supplementary material for: Emergence of mature cortical activity in wakefulness and sleep in healthy preterm and full-term infants
Source: Sleep. 2018 May 14;41(8):zsy096. doi: 10.1093/sleep/zsy096 (PMC6093466; doi:10.1093/sleep/zsy096)
Supplement: Supplementary Material [file zsy096_suppl_supplementary_results.docx]

**Supplementary Results: Non-REM sleep: slow wave EEG pattern**

The median number (inter-quartile range) of epochs included for the 37 infants in whom non-REM sleep: slow wave EEG pattern was captured was 7 (4-10).

Delta

In non-REM sleep: slow wave pattern, like non-REM sleep: tracé alternant pattern, lateral frontal, central, mid- and posterior-temporal and occipital delta power was not associated with either corrected age (CA) or postnatal age (PNA). In the tracé alternant pattern, delta power over the midline central region increased with increasing CA but this was not statistically significant for the slow wave pattern, which had a much smaller sample size (37 infants vs. 79 infants).

Theta

In non-REM sleep: slow wave pattern, like non-REM sleep: tracé alternant pattern, theta power increased with CA for every region (except the mid-temporal area) (R^2^ .129 to .207, model fit p ≤ .009; beta .359 to .456), while PNA had no effect.

Alpha-beta

In non-REM sleep: slow wave pattern, like non-REM sleep: tracé alternant pattern, lateral frontal, central, and mid- and posterior-temporal alpha-beta power was not associated with either PNA or CA. In the tracé alternant pattern, alpha-beta power over the occipital region decreased with increasing postnatal age but this was not statistically significant for the slow wave pattern, which had a much smaller sample size (37 infants vs. 79 infants).
